# The great fluid debate: saline or so-called “balanced” salt solutions?

**DOI:** 10.1186/s13052-015-0154-2

**Published:** 2015-06-25

**Authors:** Maristella Santi, Sebastiano A. G. Lava, Pietro Camozzi, Olivier Giannini, Gregorio P. Milani, Giacomo D. Simonetti, Emilio F. Fossali, Mario G. Bianchetti, Pietro B. Faré

**Affiliations:** Department of Pediatrics, Ospedale San Giovanni, 6500 Bellinzona, Switzerland; University Children’s Hospital Berne and University of Berne, 3010 Berne, Switzerland; Division of Internal Medicine and Nephrology, Ospedale Regionale, 6850 Mendrisio, Switzerland; Pediatric Emergency Department, Foundation IRCCS Ca’ Granda, Ospedale Maggiore Policlinico, 20122 Milan, Italy; Department of Internal Medicine, Ospedale San Giovanni, 6500 Bellinzona, Switzerland

**Keywords:** 0.9 % saline, Acute kidney injury, “Balanced” salt crystalloids, Hyperchloremic metabolic acidosis

## Abstract

**Background:**

Intravenous fluids are commonly prescribed in childhood. 0.9 % saline is the most-used fluid in pediatrics as resuscitation or maintenance solution. Experimental studies and observations in adults suggest that 0.9 % saline is a poor candidate for fluid resuscitation. Although anesthesiologists, intensive care specialists, perioperative physicians and nephrologists have been the most active in this debate, this issue deserves some physiopathological considerations also among pediatricians.

**Results:**

As compared with so-called “balanced” salt crystalloids such as lactated Ringer, administration of large volumes of 0.9 % saline has been associated with following deleterious effects: tendency to hyperchloremic metabolic acidosis (called dilution acidosis); acute kidney injury with reduced urine output and salt retention; damaged vascular permeability and stiffness, increase in proinflammatory mediators; detrimental effect on coagulation with tendency to blood loss; detrimental gastrointestinal perfusion and function; possible uneasiness at the bedside resulting in unnecessary administration of more fluids. Nevertheless, there is no firm evidence that these adverse effects are clinically relevant.

**Conclusions:**

Intravenous fluid therapy is a medicine like insulin, chemotherapy or antibiotics. Prescribing fluids should fit the child’s history and condition, consider the right dose at the right rate as well as the electrolyte levels and other laboratory variables. It is unlikely that a single type of fluid will be suitable for all pediatric patients. “Balanced” salt crystalloids, although more expensive, should be preferred for volume resuscitation, maintenance and perioperatively. Lactated Ringer appears unsuitable for patients at risk for brain edema and for those with overt or latent chloride-deficiency. Finally, in pediatrics there is a need for new fluids to be developed on the basis of a better understanding of the physiology and to be tested in well-designed trials.

*Omnia venenum sunt: nec sine*

*veneno quicquam existit.*

*Dosis sola facit,*

*ut venenum non fit.*^1^

*Paracelsus (1493-1541)*

## Background

Intravenous fluids are commonly prescribed in childhood. The ideal fluid achieves the desired effect with no tissue storage, no adverse electrolyte, acid-base, hematological or immunomodulating effects, is compatible with other medicines, affordable and available with long shelf life and reasonable storage requirements. However, no fluid currently meets all of these criteria [1, 2].

Fifteen years ago, it was speculated that parenteral maintenance therapy with a Na^+^-concentration lower than in blood, as proposed by Malcolm Holliday (1924–2014) in 1957, sometimes results in hyponatremic encephalopathy. The advice was followed by a controversy. In the meantime, trials comparing low-Na^+^ fluids with 0.9 % saline confirmed that traditional maintenance therapy puts patients at risk of hyponatremia [2, 3]. As a consequence, maintenance low-Na^+^ fluids are currently the exception, not the rule. Perioperative therapy with a Na^+^-content close to the physiologic range of 135-145 mmol/L has also been recommended [4].^2^

Recent studies suggest that 0.9 % saline is a poor candidate for fluid resuscitation. Although anesthesiologists, intensive care specialists, perioperative physicians and nephrologists have been the most active in this debate, this issue deserves some thoughts among pediatricians.

### 0.9 % saline – Ringer’s and Hartmann’s solutions

Saline (0.9 % NaCl solution), or physiological or normal saline (as it is frequently called), is the most-used fluid in pediatrics as resuscitation or maintenance solution and as vehicle for the administration of drugs. This is not without reason: it has been shown that saline use is equivalent and, in some circumstances, even superior to colloid solutions such as 4 % albumin or hydroxyethyl starch solutions [5].

**Fig. 1:**
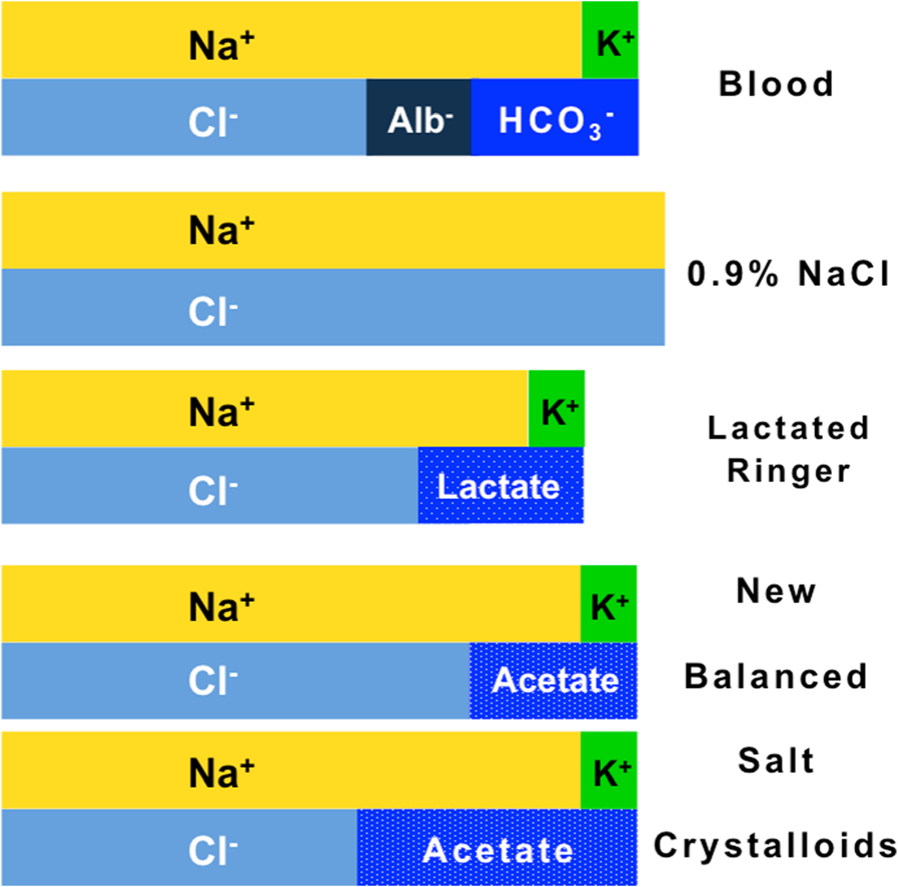
Semi-quantitative Gamble’s diagram representing the Na^+^-, K^+^-, Cl^-^-, base- and albuminate-concentration in blood, 0.9 % saline solution, lactated Ringer’s solution and so-called more “balanced” salt crystalloids. The Na^+^-concentration is higher in 0.9 % saline (≈155 mmol/L) and lower in lactated Ringer (≈130 mmol/L) than in blood (≈140 mmol/L). On the contrary, the concentration of this cation is almost identical in the “balanced” salt crystalloids and in blood (≈140 mmol/L). The Cl^-^-concentration is higher in 0.9 % saline (≈155 mmol/L), in lactated Ringer (≈110 mmol/L) and in the “balanced” salt crystalloid (≈125 mmol/L) containing acetate ≈ 25-30 mmol/L than in blood (≈100 mmol/L). The concentration of this anion is similar in the “balanced” salt crystalloid containing acetate ≈ 50 mmol/L and in blood (≈100 mmol/L). For simplicity purposes, calcium, magnesium and inorganic phosphate are omitted

Saline came into being following the ingenious in vitro studies, published in 1883, performed by the Dutch physiological chemist Hartog Hamburger (1859–1924). He noted that red blood cells were less likely to lyse in a NaCl solution of 0.9 % than in more diluted solutions [6, 7]. However, the composition of 0.9 % saline largely differs from that of blood (Fig. 1).

More physiologic solutions with a salt composition more close to that of blood than 0.9 % saline, subsequently referred to as so-called “balanced” salt crystalloids, were soon developed. Approximately 110 years ago, Sydney Ringer (1834–1910), an Australian physician, introduced a NaCl solution containing some K^+^ and Ca^++^ to promote contraction of isolated heart [6, 7]. Eighty years ago, Alexis Hartmann (1898–1964), an American pediatrician, added the non–Cl^-^ anion lactate to prevent metabolic acidosis [6, 7]. The solution developed by A. Hartmann, currently termed either Hartmann’s solution or lactated Ringer’s solution, eventually replaced the original Ringer’s solution. The composition of this solution has been slightly modified over the years (there is also some variation in its composition as supplied by different manufacturers).

### 0.9 % saline versus so-called “balanced” salt crystalloids

Large volumes of Cl^-^-rich fluids such as 0.9 % saline results in some adverse effects [6–8], as depicted in Table 1.

**Table 1:** Potentially deleterious effects of high Cl^-^-content secondary to administration of large volume of 0.9 % saline addressed in the literature

• Hyperchloremic metabolic acidosis (traditionally called dilution acidosis)	
• Acute kidney injury with reduced urine output and increase in interstitial fluid volume	
• Hyperkalemia (K^+^ mobilized from the intracellular space)	
• Damaged endothelial surface layer with increased vascular permeability and stiffness	
• Increase in proinflammatory mediators and tendency to infections	
• Detrimental effect on coagulation with tendency to blood loss	
• Detrimental gastrointestinal perfusion and function	
• Possible uneasiness at the bedside resulting in unnecessary administration of more fluids	

#### Effects on acid-base balance

Cl^-^-rich fluids containing neither HCO_3_^-^ nor another anion that can be metabolized to HCO_3_^-^, cause an increment in Cl^-^- and a decrease in HCO_3_^-^-concentration, a condition called hyperchloremic metabolic acidosis. Saline-induced acidosis, traditionally called dilution acidosis, is a short-lived phenomenon unless sustained by a kidney injury. Overall, however, the prognosis of intensive care unit patients with hyperchloremic acidosis is by far better than that of patients with non-hyperchloremic, mostly lactic metabolic acidosis [6, 7].

#### Effects on renal function

The high Cl^-^-content of saline may cause an acute kidney injury with Na^+^-retention. Following factors likely underlie this injury: 1. Increased Cl^-^-content in blood is followed by a decreased proximal tubular Cl^-^-reabsorption and thus by an increased renal tubular Cl^-^; 2. Increased renal tubular Cl^-^ results in its entry into the macula densa leading to adenosine release, which causes afferent arteriolar vasoconstriction; 3. Increased afferent arteriolar resistance reduces renal flow and glomerular filtration rate leading to reduced urine output and Na^+^-excretion. Nevertheless, although human studies confirm that large volumes of saline cause detriment to renal function, there is no firm evidence that this adverse effect is clinically relevant (this might simply reflect lack of data) [6, 7, 9].

#### Effects on potassium level

**Fig. 2:**
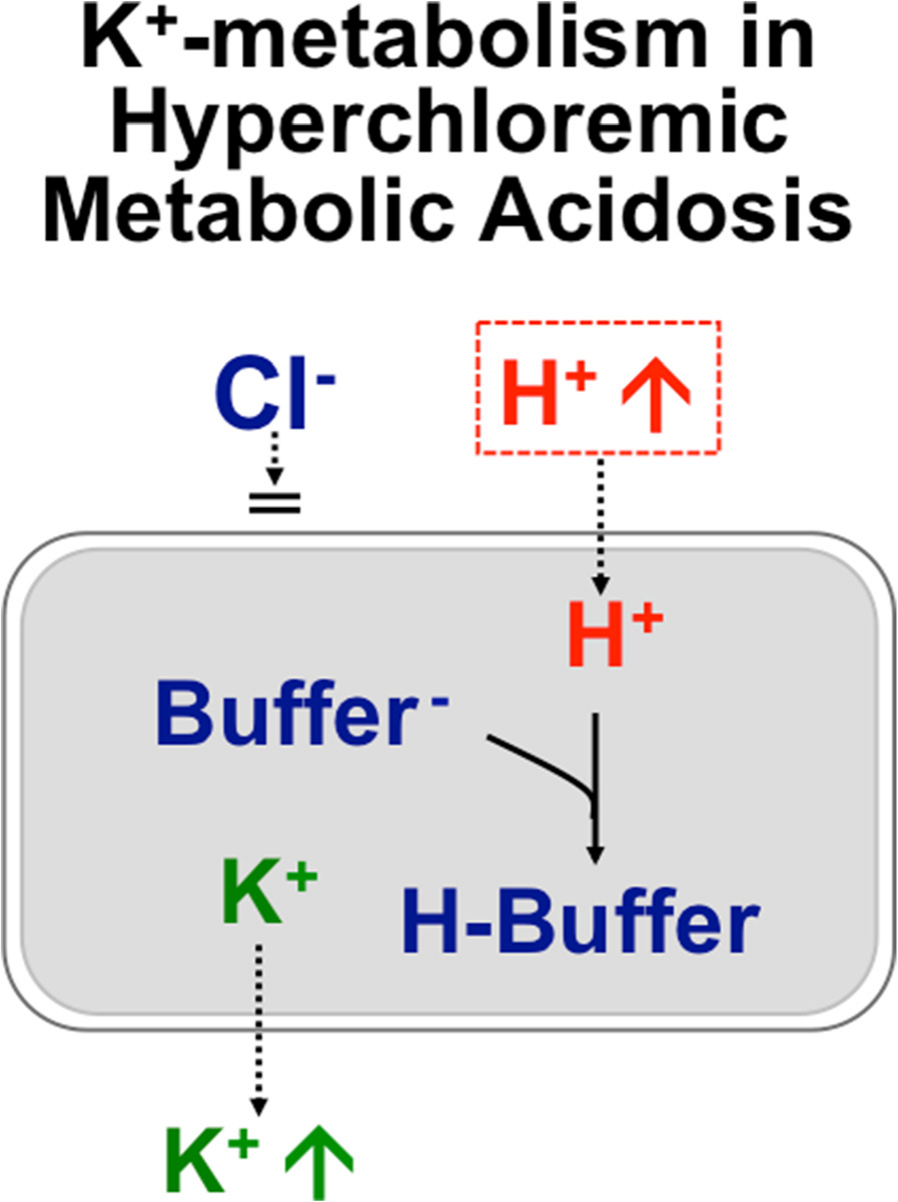
Effect of hyperchloremic metabolic acidosis on circulating K^+^ level. In hyperchloremic metabolic acidosis some extracellular H^+^ shifts into the intracellular space. Since Cl^-^ remains largely in the extracellular fluid, a shift of K^+^ from the intracellular to the extracellular fluid occurs. No tendency towards hyperkalemia occurs in normochloremic metabolic acidosis, such as lactate acidosis or ketoacidosis, because organic anions enter the intracellular fluid

As many “balanced” salt crystalloids (e.g.: lactated Ringer) contain K^+^, they are customarily contraindicated in the presence of hyperkalemia. However, the literature comparing saline with “balanced” salt crystalloids does not confirm this view. Three factors might account for this observation: a) the K^+^-content in “balanced” crystalloids of ≤5 mmol/L gets rapidly diluted in extracellular fluid after infusion; b) contrary to saline that causes hyperchloremic acidosis, “balanced” salt crystalloids do not mobilize K^+^ from the intracellular space (Fig. 2); c) kidney injury is less common in subjects infused with “balanced” salt crystalloids [6, 7].

#### Effects on vascular permeability and stiffness

**Fig. 3:**
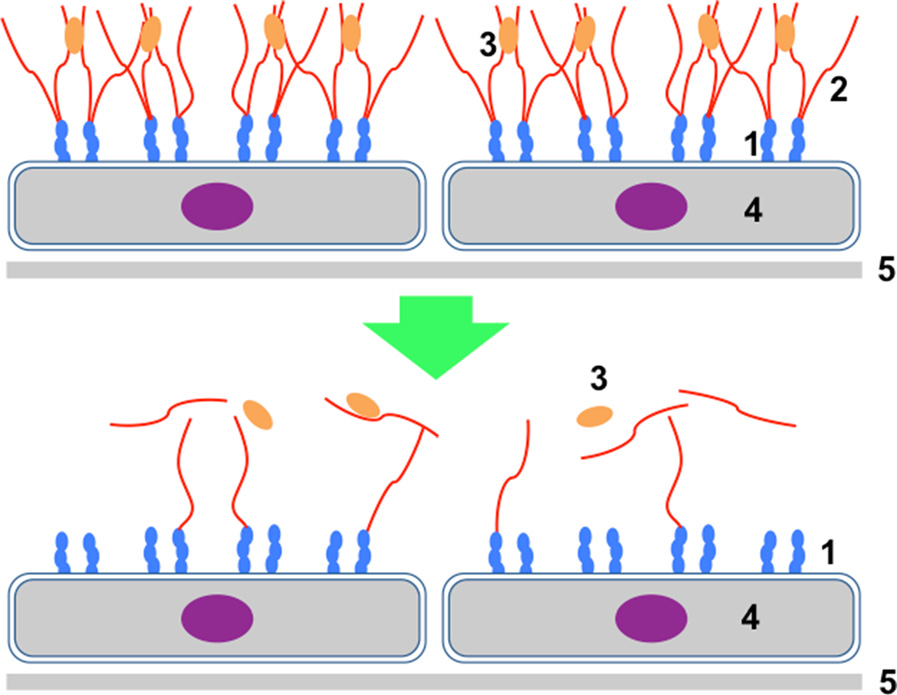
The luminal side of vascular endothelium is covered by a layer of mucopolysaccharide macromolecules of up to 1 μm thickness called endothelial surface layer or endothelial glycocalyx. This layer is the key determinant of vascular permeability. The integrity of the layer and thereby the potential for the development of interstitial edema, which varies substantially among organ systems, is often altered under inflammatory conditions when 0.9 % saline is prescribed, such as sepsis and after surgery or trauma. **1**. adhesion molecules; **2**. glycosaminoglycans; **3**. glycocalix-bound mediators; **4**. endothelial cells; **5**. endothelial basement membrane

Large volumes of saline cause a greater increase in interstitial fluid volume (and, hence, edema) than “balanced” salt crystalloids. This might reflect the aforementioned acute kidney injury with Na^+^-retention that results after saline infusion as well as an increased vascular permeability secondary to hyperchloremic metabolic acidosis. The luminal side of vascular endothelial cells is covered by a layer of mucopolysaccharide macromolecules, called endothelial surface layer or endothelial glycocalix (Fig. 3), which is the key determinant of vascular permeability. The integrity of this layer and thereby the potential for the development of interstitial edema is often altered under inflammatory conditions when salt crystalloids are commonly used, such as sepsis and after surgery or trauma (Fig. 3). Preliminary data suggest that this layer might be better preserved following resuscitation with lactated Ringer’s than with other fluids (unfortunately, no data are available with saline). Natriuretic peptides are a further factor that might damage the endothelial surface layer and perhaps also increase vascular stiffness [6, 7].

#### Inflammation

In experimental animals, the administration of saline is followed by an increase in proinflammatory mediators. Furthermore, in patients undergoing surgery, an increased risk of postoperative infections was observed in patients receiving saline compared with a “balanced” salt crystalloid [6, 7].

#### Effects on coagulation and intestine

Limited evidence suggests that large volumes of saline have a detrimental effect on coagulation as well as on gastrointestinal perfusion and function [6, 7].

#### Possible uneasiness at the bedside

Under conditions of shock, sepsis and fluid losses from the gastrointestinal tract, the acidosis that occurs with saline is frequently interpreted as a sign of hypovolemia inviting the prescription of more fluids. Consequently, the persistence of acidosis during saline administration could result in the unnecessary administration of even more fluids [7].

### More “balanced” salt crystalloids

As described above, the ionic composition of “balanced” crystalloids closely mimics the ionic make-up of the aqueous fraction of blood (Fig. 1). Unfortunately, in lactated Ringer’s solution, a commonly prescribed “balanced” solution, the Na^+^-level of ≈ 130 mmol/L is lower than in extracellular fluid (≈140 mmol/L). Consequently, this solution decreases the circulating Na^+^-level and has a potential to increase brain water content and subsequently to cause hyponatremic encephalopathy. A disproportionally large brain, a high brain-to-skull ratio, and a reduced Na^+^-pump activity notoriously put infants especially at risk of hyponatremic encephalopathy [10]. “Balanced” crystalloids with a Na^+^-level of ≈ 140 mmol/L and acetate instead of lactate were soon developed. Regrettably, little attention has been paid to the Cl^-^-concentration, which is higher in 0.9 % saline (≈155 mmol/L), lactated Ringer (≈110 mmol/L) and in the “balanced” salt crystalloid with ≈ 25-30 mmol/L of acetate (≈125 mmol/L) than in blood (≈100 mmol/L). The concentration of Cl^-^ is close to that in blood in the “balanced” salt crystalloid with ≈ 50 mmol/L of acetate.

The Na^+^/Cl^-^-ratio of 1.0 in 0.9 % saline is far lower than that of ≈ 1.4 in normal blood, denoting a tendency to cause hyperchloremic acidosis [1, 11, 12]. The Na^+^/Cl^-^-ratio is still lower than in blood in lactate Ringer (≈1.2) and in the crystalloid containing ≈ 25-30 mmol/L of acetate (≈1.1) but normal (≈1.4) in the crystalloid containing ≈ 50 mmol/L of acetate [1, 11, 12].

Lactate undergoes gluconeogenesis leading to increased levels of glucose suggesting a restrictive use in diabetes mellitus. Furthermore, administration of lactated solutions might confound the interpretation of lactate as a biomarker of tissue oxygenation, as raised lactate levels occur as a result of excessive infusion of a lactated solution. A “balanced” salt crystalloid containing acetate instead of lactate is in use since more than 50 years for severe childhood diarrhea. Acetate has some advantages over lactate: it stabilizes pH more rapidly than lactate and is metabolized in various tissues, whereas lactate is metabolized predominantly in the liver. On the other hand, acetate might possess some cardiodepressant and vasodilator effects [6, 7].

Bicarbonate, the logical alternative to lactate or acetate, is impracticable due to the difficulty in storage and its sensitivity to CO_2_. Predictably, a solution with a physiological HCO_3_^-^-concentration was withdrawn because of bicarbonate precipitations [6, 7].

### “Restricted” versus “liberal” fluid administration strategy

Finally, some recent data indicate that a restricted fluid administration strategy might be superior to a more liberal one for fluid resuscitation, for maintenance and perioperatively [3, 9–13].

## Conclusions

Intravenous fluid therapy is complex. It is a medicine like insulin, chemotherapy or antibiotics. Prescribing fluids should fit the child’s history and condition, consider the right dose at the right rate as well as the electrolyte levels and other laboratory variables. Anticipating and recognizing possible side effects and frequently assessing the patient’s condition is crucial [2].

Used generally to describe various solutions with a composition close to blood, it appears that none of the so-called “balanced” salt crystalloids is really physiological. Unsurprisingly, therefore, an ideal salt crystalloid has not been identified yet [1]. Moreover, it is unlikely that a single type of fluid, a kind of “one-size-fits-all” approach, will be suitable for all pediatric patients [2]. There is no doubt that large volumes of 0.9 % saline may impair renal function and vascular permeability. It has been therefore stated that 0.9 % saline is a “problem”, not a “solution” [14]. Hence, “balanced” salt crystalloids, although more expensive, should be preferred for volume resuscitation, maintenance and perioperatively [4, 5]. Lactated Ringer appears unsuitable for patients at risk for brain edema (e.g.: neurosurgery, head trauma, central nervous system infection) and for those with overt or latent Cl^-^-deficiency (e.g.: vomiting, salt losing renal tubular disorders, electrolyte disturbances caused by diuretics). In these two conditions, saline still remains the solution of choice [5, 10]. Obviously, in patients with fluid volume depletion associated electrolyte and acid-base abnormalities, the goal of initial management is volume resuscitation, not the repair of the associated metabolic abnormalities. Furthermore, ad hoc prepared solutions are needed in some rather rare circumstances. Yet, the process of prescribing, ordering and preparing these fluids offers several opportunities for error.

Finally, in pediatrics there is a need for novel fluids to be developed on the basis of a better understanding of the physiology and to be tested in well-designed trials. Only then will the “great fluid debate” be solved.

## Footnotes

1 All things are poison, and nothing is without poison; only the dose permits something not to be poisonous.

2 Considering the potential of hypoglycemia in infancy, 0.9% saline in 5% glucose in water (which contains approximately glucose 50 g/L and NaCl 9 g/L) is currently considered the safest composition for maintenance fluid therapy. Since perioperative administration of 5% glucose for prevention of hypoglycemia may result in stress-induced hyperglycemia, fluid therapy with a Na^+^-content close to the physiologic range with addition of 1.0-2.5% instead of 5.0% glucose is currently advised in this setting.
